# Supplementary material for: Spatial-CITE-seq: spatially resolved high-plex protein and whole transcriptome co-mapping
Source: Res Sq. 2022 Mar 30:rs.3.rs-1499315. Preprint. [Version 1] doi: 10.21203/rs.3.rs-1499315/v1 (PMC8978952; doi:10.21203/rs.3.rs-1499315/v1)
Supplement: Supplement 1 [file 855f4b0aeae0a12f1ec39c88.pdf]

Supplemental Information

Spatial-CITE-seq: spatially resolved high-plex protein and whole transcriptome co-mapping

Yang Liu[1,2], Marcello DiStasio[3], Graham Su[1,2], Hiromitsu Asashima[4,8], Archibald Enninful[1,2], Xiaoyu Qin[1,2], Yanxiang Deng[1,2], Pino Bordignon[5], Marco Cassano[5], Mary Tomayko[3,6], Mina Xu[3], Stephanie Halene[7], Joseph E. Craft[7,8,9], David Hafler[4,8,9], and Rong Fan[1,2,3,9*]

[1] Department of Biomedical Engineering, Yale University, New Haven, CT 06520, USA
[2] Yale Stem Cell Center and Yale Cancer Center, Yale School of Medicine, New Haven, CT 06520, USA
[3] Department of Pathology, Yale School of Medicine, New Haven, CT 06520, USA
[4] Department of Neurology, Yale School of Medicine, New Haven, CT 06520, USA
[5] Lunaphore Technologies SA, Route de Lully 5c, 1131 Tolochenaz, Switzerland
[6] Department of Dermatology, Yale School of Medicine, New Haven, CT 06520, USA
[7] Department of Medicine, Yale School of Medicine, New Haven, CT 06520, USA
[8] Department of Immunobiology, Yale School of Medicine, New Haven, CT 06520, USA
[9] Human and Translational Immunology Program, Yale School of Medicine, New Haven, CT 06520, USA
* to whom all correspondence should be addressed. (R.F., rong.fan@yale.edu)

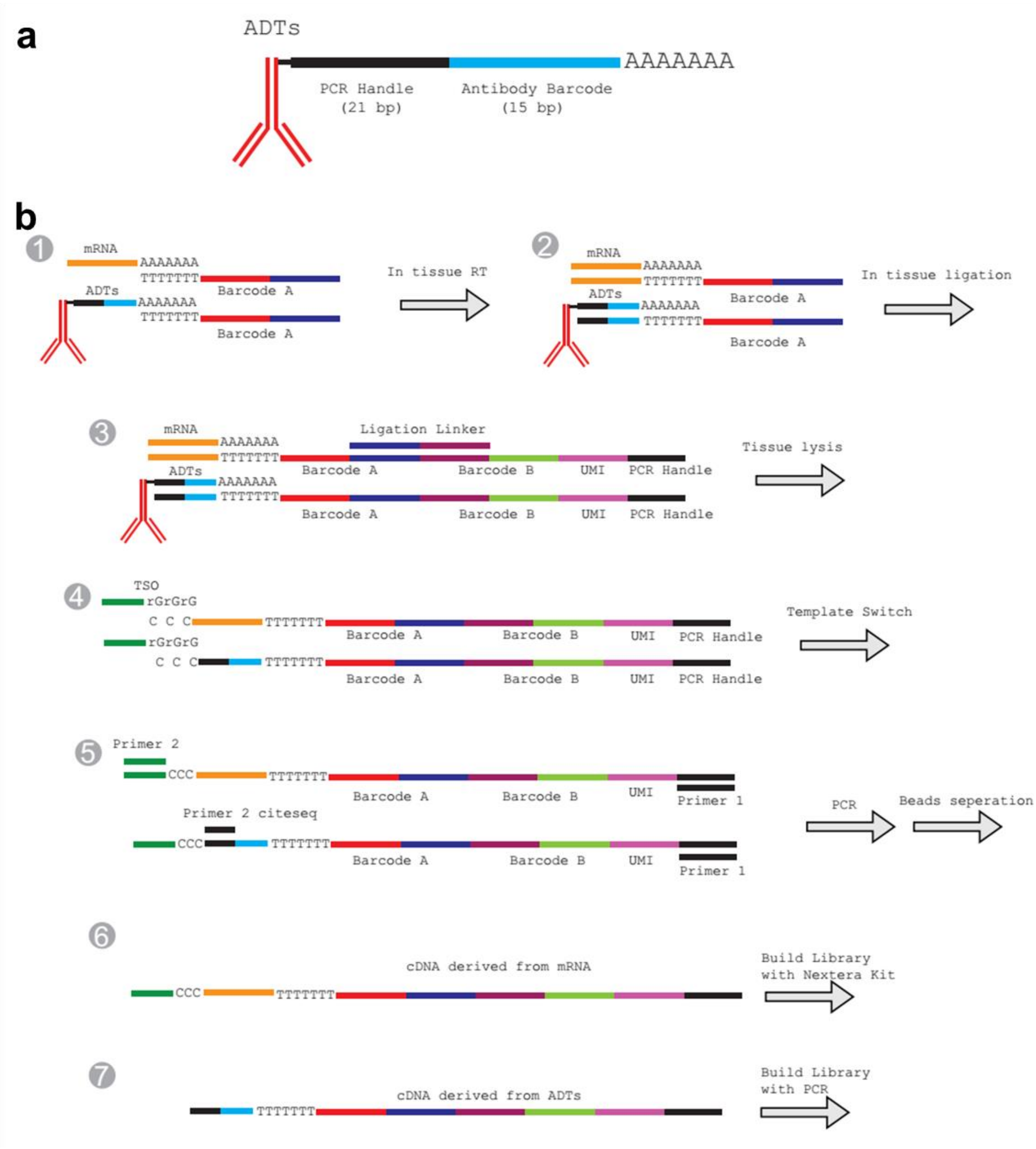

Figure S1. Spatial-CITE-seq design and detailed workflow. (a) ADT structure. The oligo labelled to the antibody has three functional regions: PCR handle (21 bp), antibody barcode (15 bp) and poly-A region (32 bp). (b) ADTs and mRNA with Poly-A region at the 3' end can be reverse transcribed into cDNA using Barcode A as the RT primer. Barcode A consists of three functional regions, the poly-T region, spatial barcode region and the ligation region. During the first flow, 50 Barcode As were loaded into 50 parallel channels and the RT reaction was carried out inside each isolated channel (Step 1&2). After peeling off the 1st PDMS, a 2nd PDMS was attached. The in-channel ligation was performed with injecting 50 Barcode Bs into each of the 50 channels which

are perpendicular to the channels of 1st PDMS chip (Step 3). Barcode B has four functional regions: ligation region, barcode region, UMI region and PCR handle region. Barcode B was also 5' biotin modification. After ligation, tissue was lysed, and cDNAs were purified with streptavidin beads. The cDNAs on the beads were templated switched with template switch oligo (Step 4). PCR was used to amplify the cDNA (Step 5). The products were split into two portions, the mRNA derived cDNAs and the ADT derived cDNAs. The library was then built separately. More details were in the method section.

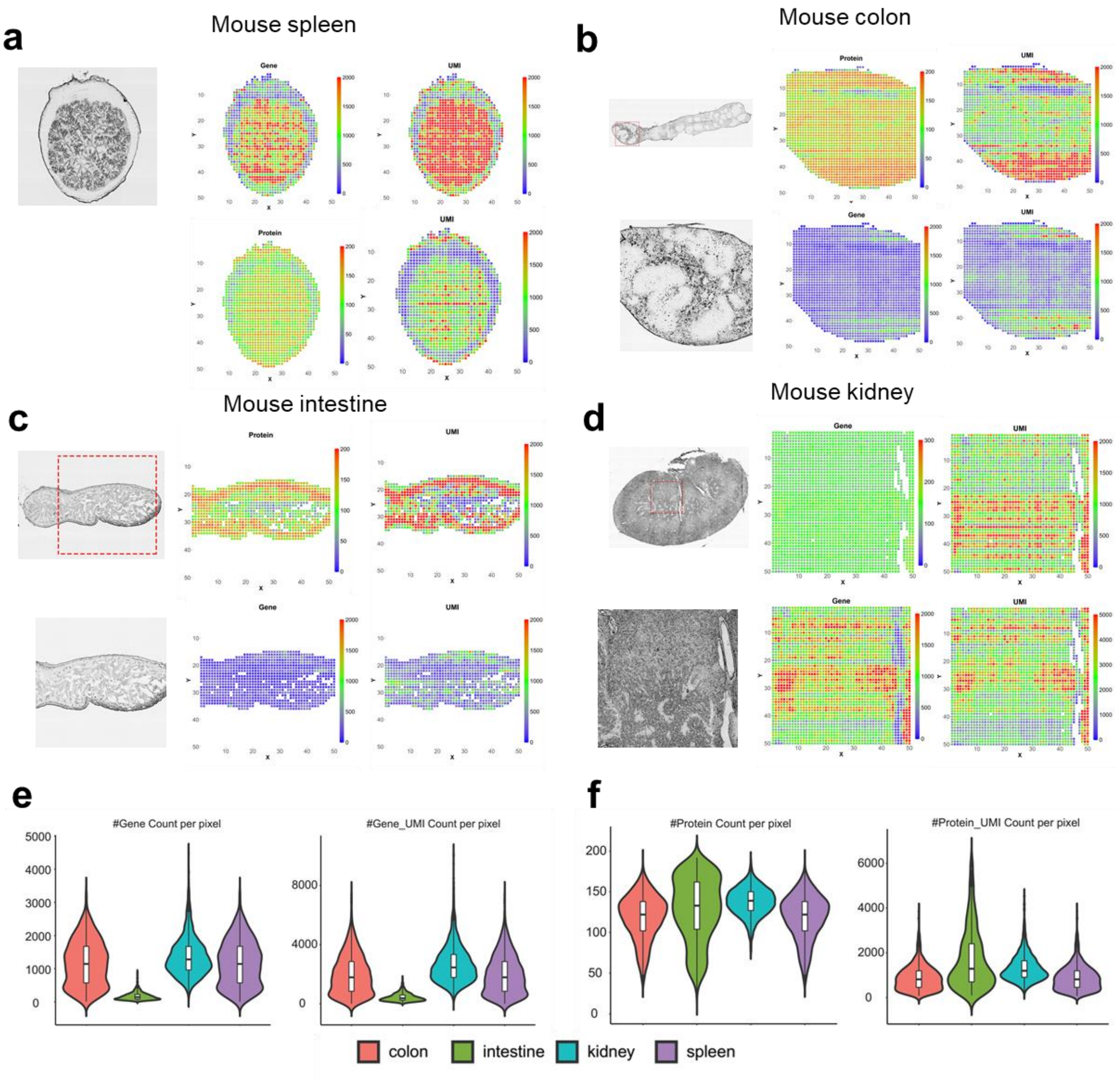

Figure S2. Spatial mapping of mouse spleen, colon, intestine and kidney with Spatial-CITE-seq. A 189 antibodies cocktail was used for all four mouse samples. The bright field image, spatial gene heatmap, spatial gene UMI heatmap, spatial protein heatmap and spatial protein UMI heatmap of spleen (a), colon (b), intestine (c) and kidney (d). (e) gene and gene UMI count per pixel of all four mouse samples. (f) Protein and protein UMI count per pixel of all four mouse samples.

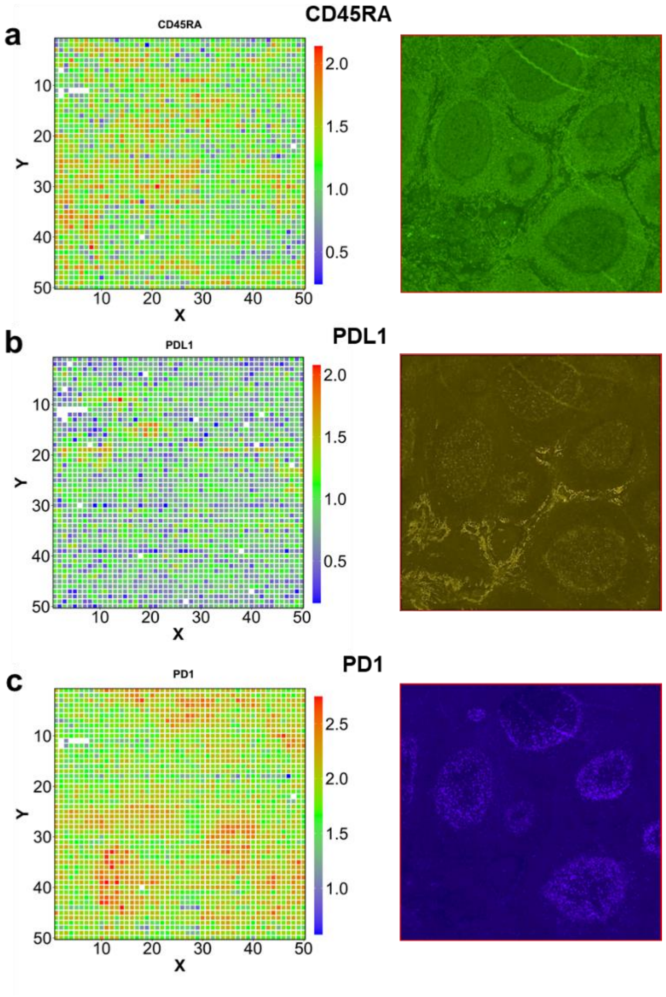

Figure S3. Immunostaining validation of spatial protein profiles. Sequential IF staining data using the FFeX technology by Lunaphore Technologies were compared side by side with Spatial-CITE-seq data. (a) CD45RA, (b) PDL1, (c) PD1. Note: the staining is not on exactly the same tonsil block.

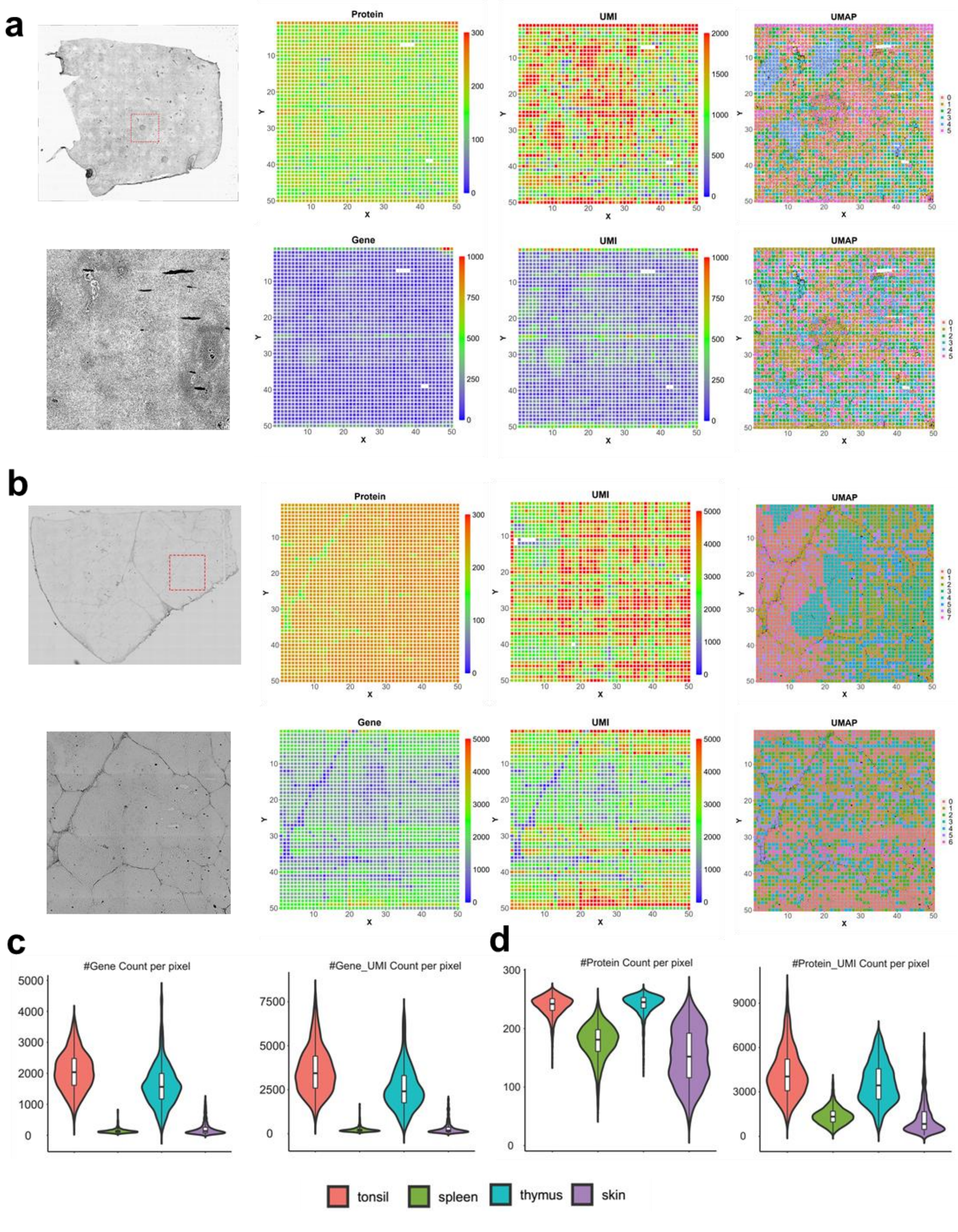

Figure S4. Spatial mapping of human spleen and thymus with Spatial-CITE-seq. A 273 antibodies cocktail was used for all four human samples. The bright field image, spatial gene heatmap, spatial gene UMI heatmap, spatial protein heatmap, spatial protein UMI heatmap, spatial clustering (based protein) and spatial clustering (based on RNA) of spleen (a) and thymus (b). (c) gene and gene UMI count per pixel of all four human samples. (d) Protein and protein UMI count per pixel of all four human samples.

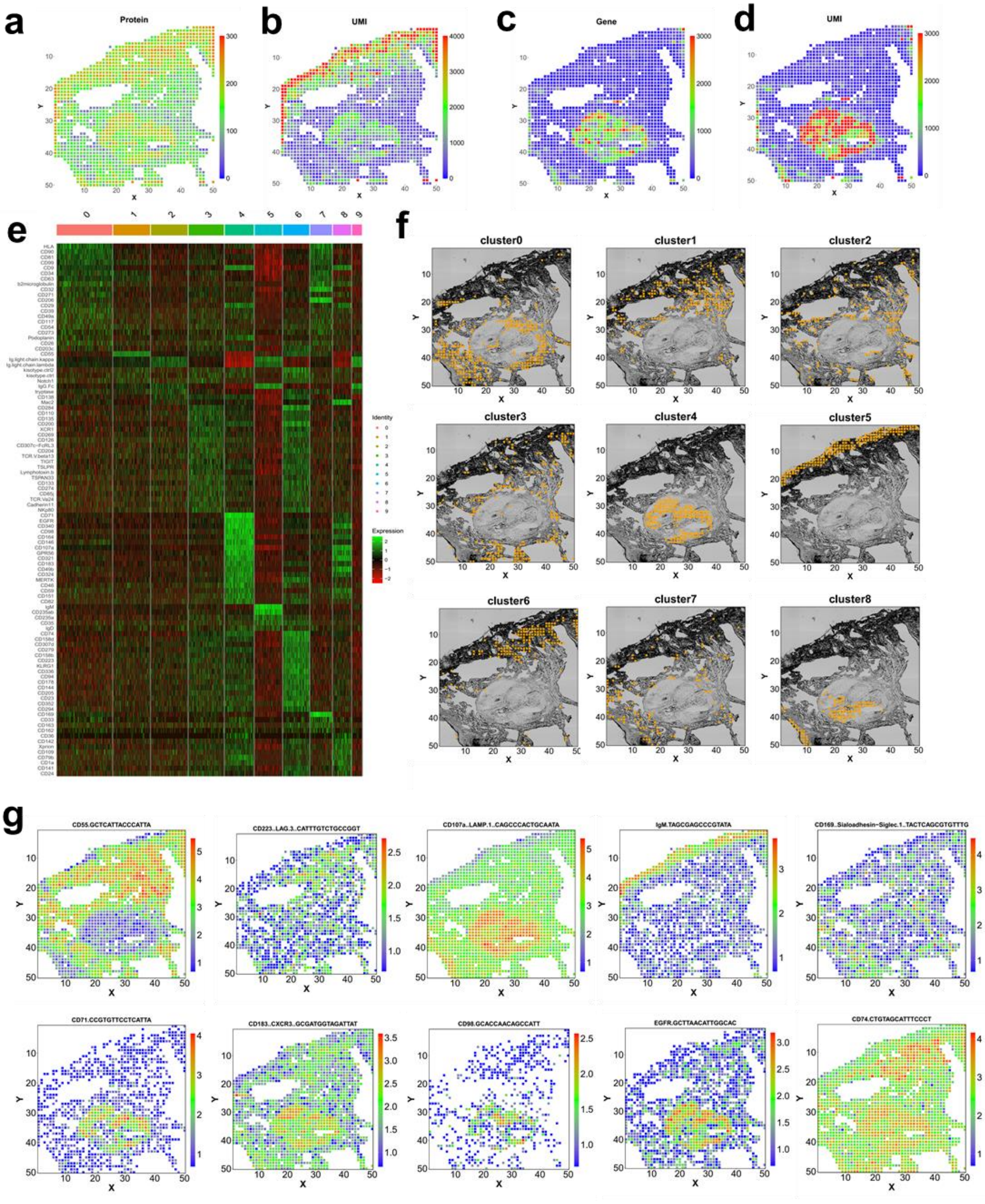

Figure S5. Spatial profiling of human skin biopsy tissue collected from the COVID-19 mRNA vaccine injection site. Spatial heatmap of gene (a), gene UMI (b), protein (c) and protein UMI (d). (e) Expression heatmap of the 10 clusters identified in skin biopsy sample. (f) the individual clusters plotted. (g) spatial distribution of some representative proteins.

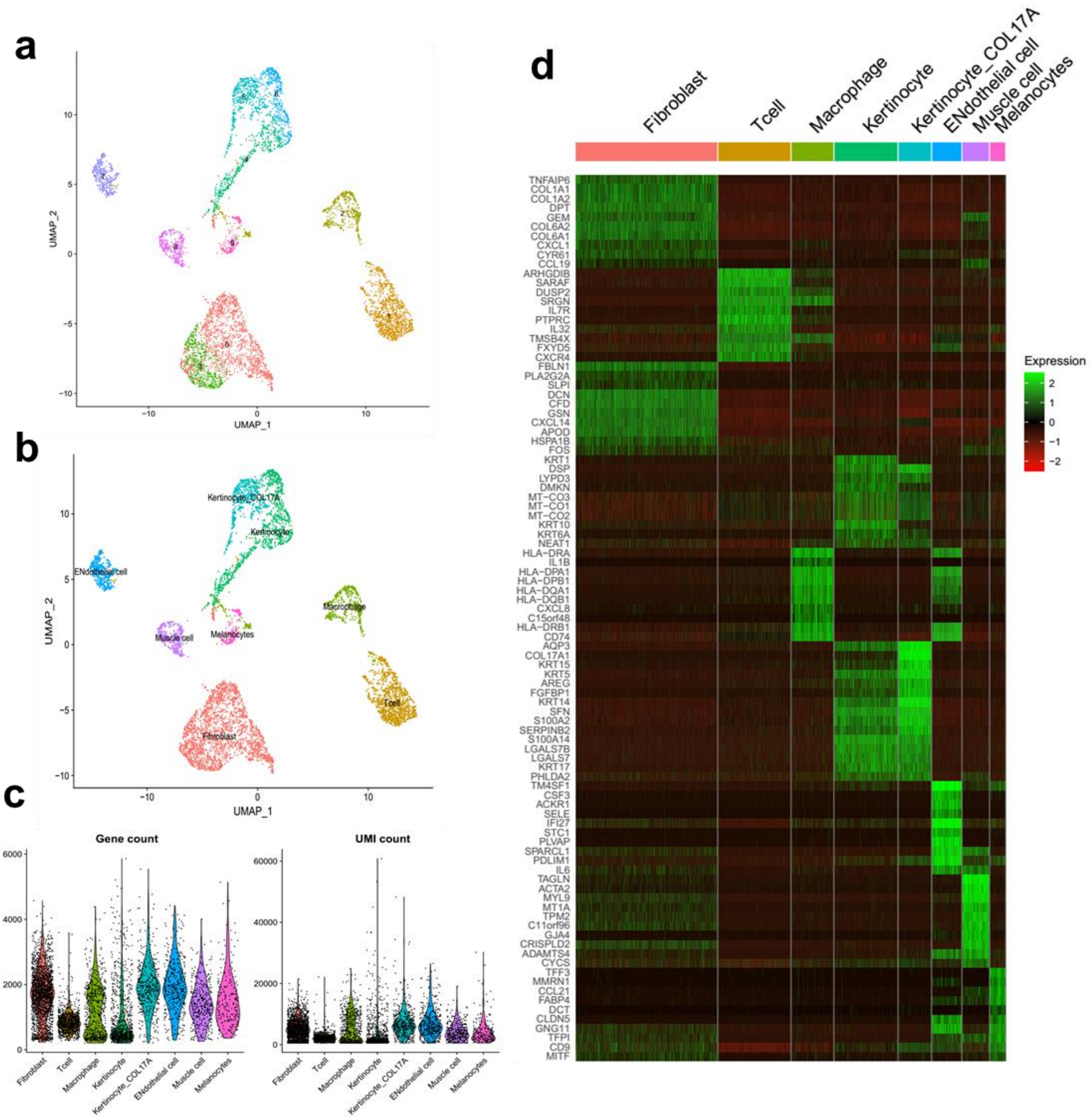

Figure S6. scRNA-seq sequencing data of skin biopsy sample. (a) spatial clusters of scRNA-seq data. (b) annotated cell types using canonical marker genes. (c) violin plot of genes and UMIs for each cell type. (d) Expression heatmap of different cell types.

Table S1. Summary of gene and protein counts for all the samples sequenced.

Sample	# Useful Pixels	# Unique genes present	# Unique proteins present	Average # genes per pixel	Average # gene UMIs per pixel	Average # proteins per pixel	Average # protein UMIs per pixel
Spleen (Mouse)	1303	19923	189	1166	1972	118	885
Colon (Mouse)	2037	19468	189	258	462	142	1213
Intestine (Mouse)	902	20444	189	172	447	129	1796
Kidney (Mouse)	2419	23750	189	1367	2675	137	1324
Tonsil (Human)	2492	28417	273	2079	3639	239	4309
Spleen (Human)	2494	20236	273	132	212	177	1403
Thymus (Human)	2500	28278	273	1647	2633	241	3700
Skin (Human)	1691	15486	273	411	815	153	1340

Table S2. DNA oligos for PCR, ligation and library preparation. All Oligos were HPLC purified.

Oligo Name	Sequence
Primer 1	CAAGCGTTGGCTTCTCGCATCT
Primer 2	AAGCAGTGGTATCAACGCAGAGT
Primer 2-citeseq	CCTTGGCACCCGAGAATT*C*C
Ligation Linker	CGAATGCTCTGGCCTCTCAAGCACGTGGAT
Template Switch Oligo	AAGCAGTGGTATCAACGCAGAGTGAATrGrG+G
N501	AATGATACGGCGACCACCGAGATCTACACTAGATCGCTCGTCGGCAG CGTCAGATGTGTATAAGAGACAG
N501-citeseq	AATGATACGGCGACCACCGAGATCTACACTAGATCGCTCGTCGGCAG CGTCAGATGTGTATAAGAGACAGCCTTGGCACCCGAGAATTCCA
N701	CAAGCAGAAGACGGCATACGAGATTCGCCTTAGTCTCGTGGGCTCGG AGATGTGTATAAGAGACAGCAAGCGTTGGCTTCTCGCATCT
N702	CAAGCAGAAGACGGCATACGAGATCTAGTACGGTCTCGTGGGCTCGG AGATGTGTATAAGAGACAGCAAGCGTTGGCTTCTCGCATCT
N703	CAAGCAGAAGACGGCATACGAGATTTCTGCCTGTCTCGTGGGCTCGG AGATGTGTATAAGAGACAGCAAGCGTTGGCTTCTCGCATCT
N704	CAAGCAGAAGACGGCATACGAGATGCTCAGGAGTCTCGTGGGCTCGG AGATGTGTATAAGAGACAGCAAGCGTTGGCTTCTCGCATCT
N705	CAAGCAGAAGACGGCATACGAGATAGGAGTCCGTCTCGTGGGCTCGG AGATGTGTATAAGAGACAGCAAGCGTTGGCTTCTCGCATCT

Table S3. The list of 50 DNA Barcode As and 50 Barcode Bs.

**Barcode A (RT primer in 1st PDMS chip)**	**Sequence**
Barcode A-1	/5Phos/AGGCCAGAGCATTCGAACGTGATTTTTTTTTTTTTTTTVN
Barcode A-2	/5Phos/AGGCCAGAGCATTCGAAACATCGTTTTTTTTTTTTTTTVN
Barcode A-3	/5Phos/AGGCCAGAGCATTCGATGCCTAATTTTTTTTTTTTTTTVN
Barcode A-4	/5Phos/AGGCCAGAGCATTCGAGTGGTCATTTTTTTTTTTTTTTVN
Barcode A-5	/5Phos/AGGCCAGAGCATTCGACCACTGTTTTTTTTTTTTTTTTVN
Barcode A-6	/5Phos/AGGCCAGAGCATTCGACATTGGCTTTTTTTTTTTTTTTVN
Barcode A-7	/5Phos/AGGCCAGAGCATTCGCAGATCTGTTTTTTTTTTTTTTTVN
Barcode A-8	/5Phos/AGGCCAGAGCATTCGCATCAAGTTTTTTTTTTTTTTTTVN
Barcode A-9	/5Phos/AGGCCAGAGCATTCGCGCTGATCTTTTTTTTTTTTTTTVN
Barcode A-10	/5Phos/AGGCCAGAGCATTCGACAAGCTATTTTTTTTTTTTTTTVN
Barcode A-11	/5Phos/AGGCCAGAGCATTCGCTGTAGCCTTTTTTTTTTTTTTTVN
Barcode A-12	/5Phos/AGGCCAGAGCATTCGAGTACAAGTTTTTTTTTTTTTTTVN
Barcode A-13	/5Phos/AGGCCAGAGCATTCGAACAACCATTTTTTTTTTTTTTTVN
Barcode A-14	/5Phos/AGGCCAGAGCATTCGAACCGAGATTTTTTTTTTTTTTTVN
Barcode A-15	/5Phos/AGGCCAGAGCATTCGAACGCTTATTTTTTTTTTTTTTTVN
Barcode A-16	/5Phos/AGGCCAGAGCATTCGAAGACGGATTTTTTTTTTTTTTTVN
Barcode A-17	/5Phos/AGGCCAGAGCATTCGAAGGTACATTTTTTTTTTTTTTTVN
Barcode A-18	/5Phos/AGGCCAGAGCATTCGACACAGAATTTTTTTTTTTTTTTVN
Barcode A-19	/5Phos/AGGCCAGAGCATTCGACAGCAGATTTTTTTTTTTTTTTVN
Barcode A-20	/5Phos/AGGCCAGAGCATTCGACCTCCAATTTTTTTTTTTTTTTVN
Barcode A-21	/5Phos/AGGCCAGAGCATTCGACGCTCGATTTTTTTTTTTTTTTVN
Barcode A-22	/5Phos/AGGCCAGAGCATTCGACGTATCATTTTTTTTTTTTTTTVN
Barcode A-23	/5Phos/AGGCCAGAGCATTCGACTATGCATTTTTTTTTTTTTTTVN
Barcode A-24	/5Phos/AGGCCAGAGCATTCGAGAGTCAATTTTTTTTTTTTTTTVN
Barcode A-25	/5Phos/AGGCCAGAGCATTCGAGATCGCATTTTTTTTTTTTTTTVN
Barcode A-26	/5Phos/AGGCCAGAGCATTCGAGCAGGAATTTTTTTTTTTTTTTVN
Barcode A-27	/5Phos/AGGCCAGAGCATTCGAGTCACTATTTTTTTTTTTTTTTVN
Barcode A-28	/5Phos/AGGCCAGAGCATTCGATCCTGTATTTTTTTTTTTTTTTVN
Barcode A-29	/5Phos/AGGCCAGAGCATTCGATTGAGGATTTTTTTTTTTTTTTVN
Barcode A-30	/5Phos/AGGCCAGAGCATTCGCAACCACATTTTTTTTTTTTTTTVN
Barcode A-31	/5Phos/AGGCCAGAGCATTCGGACTAGTATTTTTTTTTTTTTTTVN
Barcode A-32	/5Phos/AGGCCAGAGCATTCGCAATGGAATTTTTTTTTTTTTTTVN
Barcode A-33	/5Phos/AGGCCAGAGCATTCGCACTTCGATTTTTTTTTTTTTTTVN
Barcode A-34	/5Phos/AGGCCAGAGCATTCGCAGCGTTATTTTTTTTTTTTTTTVN
Barcode A-35	/5Phos/AGGCCAGAGCATTCGCATACCAATTTTTTTTTTTTTTTVN
Barcode A-36	/5Phos/AGGCCAGAGCATTCGCCAGTTCATTTTTTTTTTTTTTTVN
Barcode A-37	/5Phos/AGGCCAGAGCATTCGCCGAAGTATTTTTTTTTTTTTTTVN
Barcode A-38	/5Phos/AGGCCAGAGCATTCGCCGTGAGATTTTTTTTTTTTTTTVN

Barcode A-39	/5Phos/AGGCCAGAGCATTCGCCTCCTGATTTTTTTTTTTTTTTVN
Barcode A-40	/5Phos/AGGCCAGAGCATTCGCGAACTTATTTTTTTTTTTTTTTVN
Barcode A-41	/5Phos/AGGCCAGAGCATTCGCGACTGGATTTTTTTTTTTTTTTVN
Barcode A-42	/5Phos/AGGCCAGAGCATTCGCGCATACATTTTTTTTTTTTTTTVN
Barcode A-43	/5Phos/AGGCCAGAGCATTCGCTCAATGATTTTTTTTTTTTTTTVN
Barcode A-44	/5Phos/AGGCCAGAGCATTCGCTGAGCCATTTTTTTTTTTTTTTVN
Barcode A-45	/5Phos/AGGCCAGAGCATTCGCTGGCATATTTTTTTTTTTTTTTVN
Barcode A-46	/5Phos/AGGCCAGAGCATTCGGAATCTGATTTTTTTTTTTTTTTVN
Barcode A-47	/5Phos/AGGCCAGAGCATTCGCAAGACTATTTTTTTTTTTTTTTVN
Barcode A-48	/5Phos/AGGCCAGAGCATTCGGAGCTGAATTTTTTTTTTTTTTTVN
Barcode A-49	/5Phos/AGGCCAGAGCATTCGGATAGACATTTTTTTTTTTTTTTVN
Barcode A-50	/5Phos/AGGCCAGAGCATTCGGCCACATATTTTTTTTTTTTTTTVN
**Barcode B (Ligation to Barcode A in 2nd PDMS chip)**	**Sequence**
Barcode B-1	/5Biosg/CAAGCGTTGGCTTCTCGCATCTNNNNNNNNNNAACGTGATATC CACGTGCTTGAG
Barcode B-2	/5Biosg/CAAGCGTTGGCTTCTCGCATCTNNNNNNNNNNAAACATCGATC CACGTGCTTGAG
Barcode B-3	/5Biosg/CAAGCGTTGGCTTCTCGCATCTNNNNNNNNNNATGCCTAAATC CACGTGCTTGAG
Barcode B-4	/5Biosg/CAAGCGTTGGCTTCTCGCATCTNNNNNNNNNNAGTGGTCAATC CACGTGCTTGAG
Barcode B-5	/5Biosg/CAAGCGTTGGCTTCTCGCATCTNNNNNNNNNNACCACTGTATC CACGTGCTTGAG
Barcode B-6	/5Biosg/CAAGCGTTGGCTTCTCGCATCTNNNNNNNNNNACATTGGCATC CACGTGCTTGAG
Barcode B-7	/5Biosg/CAAGCGTTGGCTTCTCGCATCTNNNNNNNNNNCAGATCTGATC CACGTGCTTGAG
Barcode B-8	/5Biosg/CAAGCGTTGGCTTCTCGCATCTNNNNNNNNNNCATCAAGTATC CACGTGCTTGAG
Barcode B-9	/5Biosg/CAAGCGTTGGCTTCTCGCATCTNNNNNNNNNNCGCTGATCATC CACGTGCTTGAG
Barcode B-10	/5Biosg/CAAGCGTTGGCTTCTCGCATCTNNNNNNNNNNACAAGCTAATC CACGTGCTTGAG
Barcode B-11	/5Biosg/CAAGCGTTGGCTTCTCGCATCTNNNNNNNNNNCTGTAGCCATC CACGTGCTTGAG
Barcode B-12	/5Biosg/CAAGCGTTGGCTTCTCGCATCTNNNNNNNNNNAGTACAAGATC CACGTGCTTGAG
Barcode B-13	/5Biosg/CAAGCGTTGGCTTCTCGCATCTNNNNNNNNNNAACAACCAATC CACGTGCTTGAG
Barcode B-14	/5Biosg/CAAGCGTTGGCTTCTCGCATCTNNNNNNNNNNAACCGAGAATC CACGTGCTTGAG
Barcode B-15	/5Biosg/CAAGCGTTGGCTTCTCGCATCTNNNNNNNNNNAACGCTTAATC CACGTGCTTGAG
Barcode B-16	/5Biosg/CAAGCGTTGGCTTCTCGCATCTNNNNNNNNNNAAGACGGAATC CACGTGCTTGAG
Barcode B-17	/5Biosg/CAAGCGTTGGCTTCTCGCATCTNNNNNNNNNNAAGGTACAATC CACGTGCTTGAG
Barcode B-18	/5Biosg/CAAGCGTTGGCTTCTCGCATCTNNNNNNNNNNACACAGAAATC CACGTGCTTGAG

Barcode B-19	/5Biosg/CAAGCGTTGGCTTCTCGCATCTNNNNNNNNNNACAGCAGAATC CACGTGCTTGAG
Barcode B-20	/5Biosg/CAAGCGTTGGCTTCTCGCATCTNNNNNNNNNNACCTCCAAATC CACGTGCTTGAG
Barcode B-21	/5Biosg/CAAGCGTTGGCTTCTCGCATCTNNNNNNNNNNACGCTCGAATC CACGTGCTTGAG
Barcode B-22	/5Biosg/CAAGCGTTGGCTTCTCGCATCTNNNNNNNNNNACGTATCAATC CACGTGCTTGAG
Barcode B-23	/5Biosg/CAAGCGTTGGCTTCTCGCATCTNNNNNNNNNNACTATGCAATC CACGTGCTTGAG
Barcode B-24	/5Biosg/CAAGCGTTGGCTTCTCGCATCTNNNNNNNNNNAGAGTCAAATC CACGTGCTTGAG
Barcode B-25	/5Biosg/CAAGCGTTGGCTTCTCGCATCTNNNNNNNNNNAGATCGCAATC CACGTGCTTGAG
Barcode B-26	/5Biosg/CAAGCGTTGGCTTCTCGCATCTNNNNNNNNNNAGCAGGAAATC CACGTGCTTGAG
Barcode B-27	/5Biosg/CAAGCGTTGGCTTCTCGCATCTNNNNNNNNNNAGTCACTAATC CACGTGCTTGAG
Barcode B-28	/5Biosg/CAAGCGTTGGCTTCTCGCATCTNNNNNNNNNNATCCTGTAATCC ACGTGCTTGAG
Barcode B-29	/5Biosg/CAAGCGTTGGCTTCTCGCATCTNNNNNNNNNNATTGAGGAATC CACGTGCTTGAG
Barcode B-30	/5Biosg/CAAGCGTTGGCTTCTCGCATCTNNNNNNNNNNCAACCACAATC CACGTGCTTGAG
Barcode B-31	/5Biosg/CAAGCGTTGGCTTCTCGCATCTNNNNNNNNNNGACTAGTAATC CACGTGCTTGAG
Barcode B-32	/5Biosg/CAAGCGTTGGCTTCTCGCATCTNNNNNNNNNNCAATGGAAATC CACGTGCTTGAG
Barcode B-33	/5Biosg/CAAGCGTTGGCTTCTCGCATCTNNNNNNNNNNCACTTCGAATC CACGTGCTTGAG
Barcode B-34	/5Biosg/CAAGCGTTGGCTTCTCGCATCTNNNNNNNNNNCAGCGTTAATC CACGTGCTTGAG
Barcode B-35	/5Biosg/CAAGCGTTGGCTTCTCGCATCTNNNNNNNNNNCATACCAAATC CACGTGCTTGAG
Barcode B-36	/5Biosg/CAAGCGTTGGCTTCTCGCATCTNNNNNNNNNNCCAGTTCAATC CACGTGCTTGAG
Barcode B-37	/5Biosg/CAAGCGTTGGCTTCTCGCATCTNNNNNNNNNNCCGAAGTAATC CACGTGCTTGAG
Barcode B-38	/5Biosg/CAAGCGTTGGCTTCTCGCATCTNNNNNNNNNNCCGTGAGAATC CACGTGCTTGAG
Barcode B-39	/5Biosg/CAAGCGTTGGCTTCTCGCATCTNNNNNNNNNNCCTCCTGAATC CACGTGCTTGAG
Barcode B-40	/5Biosg/CAAGCGTTGGCTTCTCGCATCTNNNNNNNNNNCGAACTTAATC CACGTGCTTGAG
Barcode B-41	/5Biosg/CAAGCGTTGGCTTCTCGCATCTNNNNNNNNNNCGACTGGAATC CACGTGCTTGAG
Barcode B-42	/5Biosg/CAAGCGTTGGCTTCTCGCATCTNNNNNNNNNNCGCATACAATC CACGTGCTTGAG
Barcode B-43	/5Biosg/CAAGCGTTGGCTTCTCGCATCTNNNNNNNNNNCTCAATGAATC CACGTGCTTGAG
Barcode B-44	/5Biosg/CAAGCGTTGGCTTCTCGCATCTNNNNNNNNNNCTGAGCCAATC CACGTGCTTGAG
Barcode B-45	/5Biosg/CAAGCGTTGGCTTCTCGCATCTNNNNNNNNNNCTGGCATAATC CACGTGCTTGAG

Barcode B-46	/5Biosg/CAAGCGTTGGCTTCTCGCATCTNNNNNNNNNNGAATCTGAATC CACGTGCTTGAG
Barcode B-47	/5Biosg/CAAGCGTTGGCTTCTCGCATCTNNNNNNNNNNCAAGACTAATC CACGTGCTTGAG
Barcode B-48	/5Biosg/CAAGCGTTGGCTTCTCGCATCTNNNNNNNNNNGAGCTGAAATC CACGTGCTTGAG
Barcode B-49	/5Biosg/CAAGCGTTGGCTTCTCGCATCTNNNNNNNNNNGATAGACAATC CACGTGCTTGAG
Barcode B-50	/5Biosg/CAAGCGTTGGCTTCTCGCATCTNNNNNNNNNNGCCACATAATC CACGTGCTTGAG

Table S4. Chemicals and reagents used.

Name	Catlog Number	Vender
Maxima H Minus	EP7051	Thermo Fisher
dNTP mix	R0192	Thermo Fisher
RNase Inhibitor	Y9240L	Enzymatics
SUPERase• In™ RNase Inhibitor	AM2694	Thermo Fisher
T4 DNA Ligase	M0202L	New England Biolabs
Ampure XP beads	A63880	Beckman Coulter
Dynabeads MyOne C1	65001	Thermo Fisher
	65002	
Nextera XT DNA Preparation Kit	FC-131-1024	Illumina
Kapa Hotstart HiFi ReadyMix	KK2601	Kapa Biosystems
Proteinase K, recombinant, PCR grade	EO0491	Thermo Fisher
RNase free water	10977015	Invitrogen
Ethanol	187380-4L	Sigma
Formaldehyde solution	F8775-25ML	Sigma
Triton X-100	T8787-100ML	Sigma
NEBuffer 3.1	B7203S	New England Biolabs
T4 DNA Ligase Reaction Buffer	B0202S	New England Biolabs
Binding and washing (B&W) Buffer (2x)	15568025	Thermo Fisher
	AM9261	Thermo Fisher
	AM9760G	Thermo Fisher
Tween 20	3005	Thermo Fisher

Table S5. ADT lists for human and mouse. See attached Excel Spreadsheet - Table S5.
